# Does Tai Chi Chuan improve psychological well-being and quality of life in patients with breast cancer? Protocol for a systematic review of randomized controlled trials: A Protocol for Systematic Review and Meta-Analysis

**DOI:** 10.1097/MD.0000000000019681

**Published:** 2020-04-17

**Authors:** Jing Guo, Yifeng Shen, Bin Li, Fei Wang, Yang Jiang, Yi Lin, Jianping Chen

**Affiliations:** a Hospital of Chengdu University of Traditional Chinese Medicine; b Chengdu University of Traditional Chinese Medicine, Chengdu, Sichuan, China; c School of Chinese Medicine, University of Hong Kong, Pokfulam, Hong Kong; d Beijing Jishuitan Hospital, Xicheng District; e Guangdong Provincial Hospital of Chinese Medicine, Guangzhou, China.

**Keywords:** breast cancer, exercise, psychological well-being, quality of life, Tai Chi Chuan

## Abstract

**Background:**

Breast cancer is the most prevalent cancer in women worldwide. Treatment for breast cancer can be expensive, painful and can significantly affect the quality of life, leading to various side effects such as depression and anxiety, fatigue, sleep disorders, and cognitive impairment. Tai Chi Chuan (TCC) is the most prominent manifestation of tai chi in Chinese martial arts. TCC has been reported to be potentially effective for health and well-being of both the sick and the healthy. However, it is still controversial whether TCC benefits breast cancer patients. It is therefore of great value to evaluate the effectiveness of TCC on the psychological well-being and quality of life in people with breast cancer.

**Methods:**

This review will summarize and meta-analyze all relevant randomized controlled trials on TCC in patients with breast cancer in the light of their anxiety, depression and fatigue level, inflammatory cytokine as well as quality of life, sleep quality, and cognitive function. The following electronic databases will be searched: PubMed, Cochrane Library, EMBASE, Web of Science, China National Knowledge Infrastructure Database, Chinese Biomedical Literature Database, VIP Chinese Science and Technology Periodical Database, and Wan Fang Data. The methodologic quality of randomized controlled trials has been assessed using the Cochrane risk assessment tool. All trials included are analyzed according to the criteria of the Cochrane Handbook. Review Manager 5.3, R-3.5.1 software and grading of recommendations assessment, development, and evaluation pro-GDT online software are used for data synthesis and analysis.

**Results:**

The results of this systematic review will be used to summarize and evaluate the evidence available from randomized controlled clinical trials of TCC as supportive and adjuvant therapy for breast cancer.

**Conclusion:**

This review will provide a detailed summary of the evidence to assess the effectiveness of TCC for breast cancer.

**OSF Registration:**

DOI 10.17605/OSF.IO/Z2FSA.

## 1 Introduction

Breast cancer is the most common cancer diagnosed globally in women and accounts for about 25% of all types of cancer in women.^[1,2]^ The incidence of breast cancer is rising and the rate of mortality falling in developing countries with about 70% of deaths occurring.^[3]^ Approximately 1.4 million women are diagnosed with breast cancer worldwide and about 458,503 die of it every year.^[4]^ It is the leading cause of death among women with cancer and the 5-year survival period varies from around 40% to 80%.^[3]^ The risk of breast cancer increases with age and other risk factors, such as family history of cancer, hormonal factors, postmenopausal obesity, alcohol consumption, physical inactivity, and so on.^[5]^ Many women are diagnosed with traumatic experiences following breast cancer surgery, and common reactions such as anxiety, despair, anger, negative, and suicidal thoughts.^[6,7]^ Some treatments also cause severe long-term pain, thus affecting the cognitive function and quality of life.^[8,9]^ The diagnosis and treatment of breast cancer may also affect the women's family life, including their intimate relationship with their partners and the relationship with their offsprings.^[10,11]^ Women also have to live in the fear of recurrence of cancer and death.^[12]^ All these factors may have long-term negative effects on the mental health of breast cancer survivors. Therefore, breast cancer survivors often use complementary therapies as supportive care during cancer treatment, with the aim of strengthening health, improving quality of life, and alleviating disease symptoms and side-effects associated with conventional treatments.

Exercise is increasingly being reckoned as an effective, well-tolerated adjunct to cancer therapy and plays a role in all stages of breast cancer development, including prevention, treatment, and prognosis.^[13]^ The exercise-induced reduction in the risk of breast cancer ranges from 15% to 80%.^[14]^ There is convincing evidence that exercise reduces significantly the risk of premenopausal and postmenopausal breast cancer development and improves survival in breast cancer patients.^[15–17]^ In addition to survival benefits, exercise during cancer therapy can improve the quality of life of breast cancer patients by reducing cachexia, improving mental health, fatigue, cardiovascular functioning, body composition, bone loss, muscular strength, and flexibility.^[18,19]^

Tai Chi Chuan (TCC), as a way of life cultivation and health preservation, has become a widespread exercise worldwide. TCC has originated and developed since the 17th to 18th century in China. TCC is equivalent to a low-impact mode of aerobic exercise with slow and gentle movements associating with health benefits.^[20]^ Based on the yin and yang philosophy, TCC combines the essence of Chinese folk martial arts, art and traditional Chinese medicine theories.^[21]^ TCC is a form of movement with the concept of oriental tolerance to diversity. It cooperates with regulated breathing and meditative techniques to strengthen and relax the body and mind.^[22]^ For thousands of years, millions of Chinese people have practiced TCC to cultivate and maintain health and well-being. In recent years, TCC has gained popularity in both eastern and western countries as a characteristic and potential low-impact physical and mental exercise due to its health benefits, apparent safety, and low economic cost.

Currently TCC, as a complementary and alternative therapy of breast cancer to minimize the adverse reactions, is catching more and more attention in the relevant clinical studies. Many breast cancer patients undergoing cancer treatment exercise TCC to help manage and improve cancer-related symptoms and perimenopausal psychosomatic symptoms.^[23]^ An increasing number of clinical studies have indicated the safety and health benefits of TCC intervention for breast cancer patients. However, it is still controversial whether TCC benefits breast cancer patients on the quality of life, physical and psychological health and other important clinical endpoints. Unfortunately, the existing trial evidence is not convincing and there is currently a lack of sufficient evidence to support TCC to be an effective adjuvant supportive therapy for breast cancer.^[24]^ The goals of this study are to summarize and analyze the results of randomized controlled trials employing TCC to intervene in breast cancer and to acquire evidence of the effectiveness of TCC as supportive and adjuvant therapy for breast cancer.

## 2 Methods

This study has been registered in OSF (https://osf.io/z2fsa), and the registration number is DOI 10.17605/OSF.IO/Z2FSA. We will report this review in accordance with the preferred reporting items for systematic reviews and meta-analyses (PRISMA).^[25]^ The procedure of this protocol followed PRISMA-P guidance.^[26]^

### 2.1 Database search

Four English medical databases (Cochrane Library, PubMed, EMBASE, Web of Science) and 4 Chinese medical databases (China National Knowledge Infrastructure Database, Chinese Biomedical Literature Database, VIP Chinese Science and Technology Periodical Database, and Wan Fang Data will be systematically searched from their inceptions up to February 2020. The search strategy will be based on the guidance of the Cochrane handbook.^[27]^ The following search terms of the databases in various relevant combinations are used to screen potential studies: (breast cancer OR breast neoplasm OR breast carcinoma OR breast tumor) AND (Tai Chi OR Tai Chi Chuan OR Tai Ji OR Tai Ji Quan OR Taiji OR Tai-ji OR Taijiquan) AND (random^∗^). All relevant publications including academic dissertation and conference will be researched to ensure comprehensiveness. There will be no language and publication date restrictions.

### 2.2 Inclusion criteria

#### 2.2.1 Types of studies

Only relevant randomized controlled trials will be included.

#### 2.2.2 Types of participants

All of the participants must be female older than 18 and have been diagnosed as primary breast cancer stage 0-III patients. The main treatments the participants are currently receiving include any one of the following: surgery, radiotherapy, chemotherapy, and hormone therapy. Additionally, the participants should be under no physical limitation prohibiting exercise.

#### 2.2.3 Types of intervention

The experimental group is treated with TCC. Any type of TCC will be eligible, disregarding the styles (such as Yang-style TCC, Chen-style TCC, Wu-style TCC, Sun-style TCC) or forms (such as 24-form, 36-form, 48-form, 104-form). Comparisons can be routine treatment including standard medication and/or conventional care program, other types of standard support therapy and non-exercise therapy. All interventions will be treated for no less than 12 weeks with a frequency of at least once per week.

#### 2.2.4 Types of outcome measures

Primary Outcomes: psychological symptoms (depression and anxiety) and quality of life have been considered as the primary outcome. Secondary outcomes: the secondary outcome assessments consist of fatigue, sleep quality, cognitive function, and inflammatory cytokine. The time endpoint of the above outcomes will be no earlier than 12 weeks after initiation of treatment.

### 2.3 Exclusion criteria

(1) The unrelated and duplicated documents will be deleted.
(2) Reviews, theoretical discussions, experience summaries, and case reports will not be considered.
(3) The non-TCC type of intervention and articles without the original data will be not be included.

### 2.4 Data collection and extraction

Study screening and data extraction were based on the Cochrane collaborative network system evaluator handbook.^[27]^ NoteExpress (version: 3.2, Beijing Aegean Software Company) software for literature import and screening was used to exclude duplicate literature, and to exclude unrelated literature by reading the titles and abstracts. Full texts that retain the randomized clinical trials meeting the inclusion criteria will be read. Two reviewers (GJ and SYF) will extract the data independently by adopting a self-developed data extraction form. Any disagreement in the process will be resolved by discussion with another team member (LB).

The following characteristics and data from each included study will be extracted. The general information will include research ID (primary author, year of publication, country of origin), title, publication status, report sources, fund support. Methodology information will include the design, number of arms, random sequence generation, allocation concealment, blinding, incomplete outcome data, selective reporting, sample size calculation, baseline comparability. For participant information, it will include the diagnostic criteria, inclusion criteria, exclusion criteria, sample size, age, population, and status of cancer. Intervention information will include the type of intervention, current treatment, duration and frequency of treatment, intervention instructor and patient follow-up (reasons and number of patients who dropped out or got lost). Inclusion of all relevant outcome indicators ensures a comprehensive coverage.

**Figure 1:**
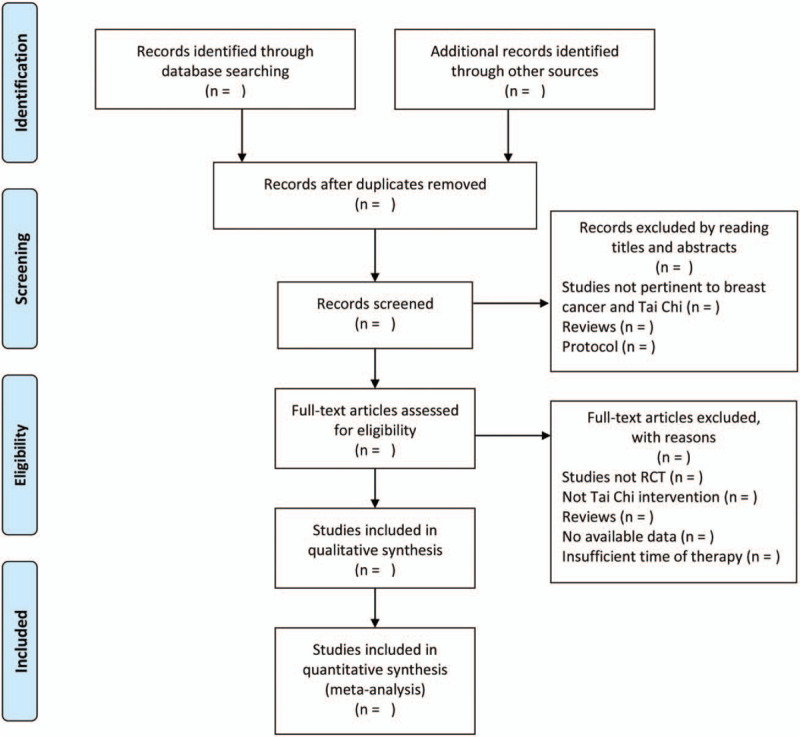
Flow diagram of the study selection process.^[42]^ Arrows = flow directions or reasons for exclusion of trials, RCT = randomized controlled trial, TCC = Tai Chi Chuan.

The selection process is shown in a PRISMA flow chart (http://www.prisma-statement.org/) (Fig. 1).

### 2.5 Assessment of methodological quality

Risk of bias will be assessed by the Cochrane risk assessment tool in the following domains^[27]^: random sequence generation, allocation concealment, blinding of the participants and personnel, blinding of outcomes assessment, incomplete outcome data, selective outcome reporting, and other bias. Two reviewers (GJ and SYF) will independently assess the risk of bias for each included study. The low risk of bias, high risk of bias, and unclear risk of bias will be used as a code for the evaluations of the above 7 domains. Disagreements will be resolved by discussion. If necessary, we will contact the authors of the included studies to get some missing information. Trials with a high risk of bias will be considered for sensitivity analysis.

### 2.6 Data synthesis and analysis

Review Manager Software (RevMan, Version 5.3 for Windows, The Cochrane Collaboration, Oxford, England) will be applied to analyze and synthesize the outcome measures systematically. The quantitative synthesis will be conducted on the outcomes when clinical heterogeneity is not considered by at least 2 reviewers in a discussion and qualitative description is adopted if clinical heterogeneity exists. Continuous variable is described by mean difference, *P-*value and 95% confidence interval. The relative risk, *P-*value and 95% confidence interval are used to describe the dichotomous outcomes. *I*^2^ test is used to judge the heterogeneity of meta-analysis. *I*^2^ value >50% is considered as significant heterogeneity. In this case, the data will be analyzed using a random-effect model. Otherwise, the fixed-effect model should be adopted. Sensitivity analysis is adopted to ensure the stability results by eliminating low-quality trials. If the necessary data are available, the grouping factor for subgroup analysis will be done for different duration or frequency of TCC intervention.

### 2.7 Publication bias

The potential publication bias will be analyzed by the funnel plot or Egger test. Funnel plot will be used if the number of trials included in the meta-analysis is no less than 10. Otherwise, the Egger test should be applied. The analysis software is R 3.5.1 for Windows.

### 2.8 Quality of evidence

This study evaluates the evidence of clinical outcomes according to grading of recommendations assessment, development, and evaluation standard with consideration of the many factors that could reduce the quality of evidence, such as limitations in study design, inconsistencies in results, discontinuity in evidence, inaccuracy, and publication bias. Grading of recommendations assessment, development, and evaluation Pro GDT online software will be used to form the summary of findings table (SoF table).

## 3 Discussion

As complementary and alternative therapy, TCC has become a popular fitness exercise worldwide. What kind of person is TCC suitable for? TCC can be practiced by anyone who wishes to improve physical and mental functions. Specifically, it has been shown to benefit patients with breast cancer, chronic heart failure, rheumatoid arthritis, and stroke.^[28]^ Breast cancer is the most common cancer in women. Treatment for breast cancer can be disfiguring, expensive and painful and can significantly affect the quality of life, leading to various side effects such as depression and anxiety, fatigue, sleep disorders, and cognitive impairment.^[29]^ Being diagnosed with breast cancer means dealing with many stressors, including side effects of treatment, fear of cancer recurrence, fear of death, impaired body image, and financial burden.^[30]^ Studies have shown that nearly half the cancer patients develop depressive symptoms during treatment, and the rate is particularly high among breast cancer patients, at least twice as high as those without breast cancer.^[31,32]^ Depression and anxiety are the most common mental disorders affecting patients after breast cancer surgery and are one of the major predictors of quality of life. This mood disorder is highly prevalent for 12 to 24 months after diagnosis and treatment of breast cancer and may be associated with increased mortality.^[33–35]^ Fatigue is one of the most common and distressing symptoms experienced by breast cancer patients and causes significant impairment in QOL.^[36]^ Psychological factors are strongly associated with breast cancer-related fatigue and can increase a woman's risk of experiencing fatigue after treatment and in subsequent years. There has been growing interest in the relationship between sleep disorders and breast cancer, as well as in treatment. Recent evidences have shown a significant correlation between sleep disorder and all-cause mortality in breast cancer patients.^[37,38]^ And sleep disorders were associated with a 1.3-fold increase in the risk of breast cancer metastasizing within 5 years of the initial diagnosis.^[39]^ In addition, cancer-related cognitive impairment is a significant problem in breast cancer patients, and it negatively impacts QOL.^[40]^ However, its trajectory is not fully understood and this clinically important outcome has not received much attention. Overall, all of these cancer-related clinical outcomes have a greater or lesser impact on the QOL and psychology of breast cancer patients. Currently, there is a need for clinical treatment programs to deal with these adverse manifestations of breast cancer.

Nowadays, TCC is particularly popular among women with breast cancer, many of them practice it to help combat adverse reactions after breast cancer surgery and perimenopausal psychosomatic symptoms. However, from the systematic reviews that have been reported so far, TCC, as an adjuvant and supportive treatment for breast cancer, has not shown convincing and consistent evidence to improve the related adverse reactions in patients.^[20,41]^ There is a clear need to better demonstrate the efficacy of TCC in the treatment of breast cancer. This systematic review will focus on the effects of TCC on their anxiety, depression and fatigue level, inflammatory cytokine as well as quality of life, sleep quality, and cognitive function in breast cancer patients. Results from this study will be valuable for clinical practice and research on TCC and breast cancer. Shortcomings of the existing studies will be found to provide implications for future high-quality clinical trials and to get explicit evidence on TCC for breast cancer. We hope this review will stimulate the proper evaluation of TCC.

## Author contributions

**Performed risk of bias assessment:** Jing Guo, Yifeng Shen, and Bin Li.

**Software:** Jing Guo, Yifeng Shen and Yang Jiang.

**Supervision, revision article:** Jianping Chen.

**Writing – original draft:** Jing Guo and Yifeng Shen.

**Writing – review & editing:** Jing Guo, Yifeng Shen, and Bin Li.

Yifeng Shen orcid: 0000-0003-0356-1420.

Bin Li orcid: 0000-0002-7123-6286.

Jing Guo orcid: 0000-0001-9861-0250.
